# Editorial: Cell-based therapeutics for intervertebral disc degeneration

**DOI:** 10.3389/fbioe.2023.1250266

**Published:** 2023-07-06

**Authors:** Zhen Li, Lachlan J. Smith, Bin Li

**Affiliations:** ^1^ AO Research Institute Davos, Davos, Switzerland; ^2^ Department of Orthopaedic Surgery, University of Pennsylvania, Philadelphia, PA, United States; ^3^ Department of Orthopaedic Surgery, The First Affiliated Hospital, School of Biology & Basic Medical Sciences, Suzhou Medical College, Orthopedic Institute, Soochow University, Suzhou, Jiangsu, China

**Keywords:** intervertabral disc, biomaterials, drug delivery, nucleus pulposus, small molecules, stem cells, cell therapy

## Introduction

Low back pain (LBP) is the leading cause of disability worldwide, and represents an enormous social and economic burden. One of the main causes of LBP is intervertebral disc (IVD) degeneration (IVDD). The causes of IVDD are complex and include genetic, nutritional, metabolic, and mechanical factors. While the pathogenesis of IVDD has been extensively studied, treatment options are still limited. When conservative therapies fail, surgical interventions may have to be performed; however, none of these therapies are able to repair the damaged tissue and revive a functional IVD. In recent decades, substantial progress has occurred in tissue engineering and cell-based therapies. Several cell types including mesenchymal stem cells (MSCs), chondrocytes, and other progenitor cells have been investigated in laboratory studies, and a number of clinical trials have commenced, providing new hope for IVDD treatment and patients with LBP. This current Research Topic represents a collected series of articles in this field. Highlights are summarized below.

## Research topic highlights

Intradiscal implantation of MSCs has shown promising results for IVD regeneration in preclinical and clinical studies; however, the survival and differentiation fate of MSCs after transplantation are still unclear. Recent studies revealed that extracellular vesicles (EVs) secreted by MSCs contain key molecules associated with the immunomodulatory and regenerative effects of these cells. Xia et al. provided a review describing the application of MSC-derived EVs for IVD regeneration. Studies on EVs from various MSC sources, induced pluripotent stem cells, and IVD derived progenitor cells were summarized. The study of Tilotta et al. compared the effect of secretomes from bone marrow and adipose tissue-derived MSCs on human nucleus pulposus (NP) cells *in vitro*. Both secretomes improved the proliferative capacity, viability, and glycosaminoglycan synthesis of human NP cells in basal conditions and following pro-inflammatory preconditioning with IL-1β. Bone marrow-derived MSC secretomes exerted a greater regenerative effect compared to adipose tissue-derived MSC secretomes, as indicated by gene expression data, which further highlights the importance of selecting the right cell source for EV treatment.

Another strategy to improve the therapeutic efficacy of MSCs for IVDD is co-delivery of bioactive factors to enhance the performance of these cells in the degenerate IVD microenvironment. Hu et al. investigated whether co-delivery of salvianolic acid B (SalB), a bioactive compound extracted from the Chinese herb salvia miltiorrhiza, could improve MSC survival and function in the degenerate IVD. *In vitro*, SalB was found to reduce oxidative stress-induced apoptosis of MSCs and enhance proteoglycan production. *In vivo*, using a rat caudal IVD puncture model, co-delivery of SalB with MSCs in a hyaluronic acid hydrogel carrier was found to be more effective at preventing progression of degeneration than MSCs or hydrogel alone, supporting the contention that composite therapeutic approaches may result in the best treatment outcomes.

A promising alternative strategy to injection of exogenous, therapeutic cells to regenerate the IVD is delivery of anabolic agents to target and rejuvenate endogenous, degenerate IVD cells. Wang et al. investigated the use of scutellarin, a bioactive ingredient extracted from the Chinese flowering plant erigeron breviscapus, as a potential anti-inflammatory therapy for IVDD. In human cell culture experiments, scutellarin was found to suppress the catabolic effects of tumor necrosis factor-alpha (TNF-α). *In vivo*, locally-delivered scutellarin was found to ameliorate IVDD progression in a rat caudal IVD puncture model. In advancing this and other similar promising therapies towards clinical translation, a challenge will be identifying the most effective delivery route to ensure that therapeutic compounds can have a sustained, local therapeutic effect on the IVD.

To address this challenge, recently, treatment strategies based on microspheres, which have been widely used as carriers for targeted drug delivery and controlled release, have brought new hope for IVD regeneration. The review article by Guo et al. discusses the possible mechanisms of IVDD and the limitations of current treatments, focusing on the application of microsphere-based drug delivery for treating IVDD. Advantages of microspheres include excellent injectability and biocompatibility. IVD regeneration requires mobilization of endogenous factors and regulation of the local microenvironment. Microspheres can be used to deliver drugs and bioactive substances to promote IVD regeneration. As the basis of tissue regeneration, cells can also be loaded on microspheres and be delivered to the target site.

The treatment effects for IVDD remain limited because they cannot reverse degenerative changes and restore healthy IVD function. During the etiology of IVDD, the level of reactive oxygen species (ROS), caused by mechanical stress, inflammatory cytokines, nutritional deficiency and aging, significantly increases. Mitochondria are not only the main source of ROS, but are also vulnerable to oxidative damage. Excessive ROS can trigger oxidative stress, lead to mitochondrial dysfunction and activate endoplasmic reticulum stress, and finally lead to a cascade reaction that triggers apoptosis and cell death. Therefore, finding a suitable method to eliminate oxidative stress induced by mitochondrial dysfunction of NP cells may be a potential therapeutic strategy for IVDD. The study by Zhang et al. showed that hyaluronic acid (HA) has many pharmacological effects, such as antioxidation, anti-inflammation, analgesia, anti-apoptosis and inhibition of extracellular matrix (ECM) degradation. As a result, HA protects mitochondrial function by activating mitochondrial phagocytosis of NP cells, and thus ameliorates IVDD. Further, it was also found that HA treatment induces mitophagy in NP cells via the C1QBP signaling pathway under oxidative stress conditions. In addition, a bovine IVD organ culture model will likely be equipped shortly to further prove the therapeutic potential of HA. In summary, findings from this study support the potential of HA for treating IVDD.

Collectively, the articles in this Research Topic highlight innovative, emerging strategies for treating IVDD, providing hope for patients with debilitating LBP.

